# Accurate splice site prediction using support vector machines

**DOI:** 10.1186/1471-2105-8-S10-S7

**Published:** 2007-12-21

**Authors:** Sören Sonnenburg, Gabriele Schweikert, Petra Philips, Jonas Behr, Gunnar Rätsch

**Affiliations:** 1 Fraunhofer Institute FIRST, Kekuléstr. 7, 12489 Berlin, Germany; 2 Friedrich Miescher Laboratory of the Max Planck Society, Spemannstr. 39, 72076 Tübingen, Germany; 3 Max Planck Institute for Biological Cybernetics, Spemannstr. 38, 72076 Tübingen, Germany; 4 Max Planck Institute for Developmental Biology, Spemannstr. 35, 72076 Tübingen, Germany

## Abstract

**Background:**

For splice site recognition, one has to solve two classification problems: discriminating true from decoy splice sites for both acceptor and donor sites. Gene finding systems typically rely on Markov Chains to solve these tasks.

**Results:**

In this work we consider Support Vector Machines for splice site recognition. We employ the so-called *weighted degree *kernel which turns out well suited for this task, as we will illustrate in several experiments where we compare its prediction accuracy with that of recently proposed systems. We apply our method to the *genome-wide *recognition of splice sites in *Caenorhabditis elegans*, *Drosophila melanogaster*, *Arabidopsis thaliana*, *Danio rerio*, and *Homo sapiens*. Our performance estimates indicate that splice sites can be recognized very accurately in these genomes and that our method outperforms many other methods including *Markov Chains*, *GeneSplicer *and *SpliceMachine*. We provide genome-wide predictions of splice sites and a stand-alone prediction tool ready to be used for incorporation in a gene finder.

**Availability:**

Data, splits, additional information on the model selection, the whole genome predictions, as well as the stand-alone prediction tool are available for download at .

## Introduction

With the generation of whole genome sequences, important insight into gene functions and genetic variation has been gained over the last decades. As novel sequencing technologies are rapidly evolving, the way will be paved for cost efficient, high-throughput whole genome sequencing which is going to provide the community with massive amounts of sequences. It is self-evident that the handling of this wealth of data will require efficient and accurate computational methods for sequence analysis. Among the various tools in computational genetic research, gene prediction remains one of the most prominent tasks, as recent competitions have further emphasised (e.g. [1,2]). Accurate gene prediction is of prime importance for the creation and improvement of annotations of recently sequenced genomes [3,4]. In the light of new data related to natural variation (e.g. [5-7]), the importance of accurate computational gene finding gains increasing importance since it helps to understand the effects of polymorphisms on the gene products.

*Ab initio *gene prediction from sequence is a highly sophisticated procedure as it mimics – in its result – the labour of several complex cellular machineries at a time: identification of the beginning and the end of a gene, as is accomplished by RNA polymerases; splicing of the nascent RNA, in the cell performed by the spliceosome; and eventually the detection of an open reading frame, as does the ribosome. The success of a gene prediction method therefore relies on the accuracy of each of these components. In this paper we will focus on the improvement of signal sensors for the detection of splice sites, as this sub-problem is a core element of any gene finder. A comprehensive understanding of splice sites is not only a prerequisite for splice form prediction but can also be of great value in localizing genes [8-12].

In eukaryotic genes, splice sites mark the boundaries between exons and introns. The latter are excised from premature mRNAs in a post-processing step after transcription. Both the donor sites at the exon-intron junctions, and the acceptor sites at the intron-exon boundaries, have quite strong consensus sequences which can, however, vary significantly from one organism to another. The vast majority of all splice sites are so called *canonical splice sites *which are characterised by the presence of the dimers GT and AG for donor and acceptor sites, respectively. The occurrence of the dimer is not sufficient for the splice site. Indeed, it occurs very frequently at non splice site positions. For example in human DNA, which is ≈6·10^9 ^nucleotides in size, GT can be found about 400 million times (summed over both strands). For some crude estimate of say 2·10^4 ^genes with 20 exons each, only 0.1% of the consensus sites are true splice sites. We therefore face two extremely unbalanced classification tasks, namely the discrimination between true donor sites and decoy positions with the consensus dimer GT or GC (the only non-canonical splice site that we will consider) and the discrimination between true acceptor sites and decoy positions with the consensus dimer AG.

### Relation to previous work

Although present-day splice site detectors (e.g. based on Support Vector Machines, neural networks, hidden Markov models) are reported to perform at a fairly good level [9,13-15], several of the reported performance numbers should be interpreted with caution, for a number of reasons. First of all, these results are based on *small *and potentially biased data sets. Now that many genomes have been fully sequenced, these results will need to be re-evaluated. Second, issues in generating negative examples (decoys) were, if recognized, often not sufficiently documented. The choice of data sets, in particular the decoys, can make a tremendous difference in the measured performance. Third, often only the single site prediction of acceptor and donor sites is considered, whereas the higher goal is to use the splice site predictor within a gene finder. It is uncertain how good the predictors perform in this setting. Keeping these in mind, we provide unbiased *genome-wide *splice site prediction which enables further evaluation in gene finders.

In this paper, we will apply Support Vector Machines (SVMs) to the recognition of splice sites. SVMs are known to be excellent algorithms for solving classification tasks [16-19], and have also been successfully applied to several bioinformatics problems [3,20-23] including splice site detection, cf. e.g. [24-32]. Our work builds upon our previous work: In [24,25] we demonstrated that SVMs using kernels from probabilistic hidden Markov models (cf. [20,23]) outperform hidden Markov models *alone*. As this approach did not scale to many training examples, we performed a comparison of different *faster *methods for splice site recognition [28], where we considered Markov models and SVMs with different kernels: the so-called *locality improved kernel*, originally proposed for recognition of translation initiation sites [21]; the *SVM-pairwise kernel*, using alignment scores [33]; the *TOP kernel*, making use of a probabilistic model (cf. [20,23]); the standard *polynomial kernel *[16]; and the so-called *weighted degree kernel *[28,34]. A predictor based on the latter kernel has been successfully used in combination with other information for predicting the splice form of a gene, while outperforming other HMM based approaches [3]. This indicates that the improved accuracy of splice site recognition indeed leads to a higher accuracy in *ab initio *transcript prediction.

Other groups also reported successful SVM based splice site detectors. In [27] it was proposed to use linear SVMs on binary features computed from di-nucleotides, an approach which also outperformed previous Markov models. Even more accurate, the authors of SpliceMachine [29] not only used positional information (one- to trimers) around the splice site, but also explicitly modelled compositional context using tri- to hexamers. To the best of our knowledge, this approach is the current state-of-the art, outperforming previous SVM based approaches as well as GeneSplicer [12] and GeneSplicerESE [35]. In [31] linear SVMs were used on positional features that were extracted from empirical estimates of unconditional positional probabilities. Note that this approach is similar to our TOP kernel method on zeroth-order Markov chains [28]. Recently, [32] reported improved accuracies for splice site prediction also by using SVMs. The method employed in [32] is very similar to a kernel initially proposed in [21] (*Salzberg kernel*). The idea of this kernel is to use empirical estimates of conditional positional probabilities of the nucleotides around splice sites (estimated by Markov models of first order) which are then used as input for classification by an SVM.

Many other methods have been proposed for splice site recognition. For instance multilayer neural networks with Markovian probabilities as inputs [15]. They train three Markov models on three segments of the input sequence, the upstream, signal and downstream segments. Although they outperform [32] on small datasets, the authors themselves write that the training of the neural networks is especially slow when the number of true and decoy examples are imbalanced and that they have to downsample the number of negatives for training even on small and short sequence sets. Therefore, their method does not seem suitable for large-scale genome-wide computations. Finally, [36] proposed a method based on Bayesian Networks which models statistical dependencies between nucleotide positions.

In this work we will compare a few of our previously considered methods against these approaches and show that the engineering of the kernel, the careful choice of features and a sound model selection procedure are important for obtaining accurate predictions of splice sites.

Our previous comparison in [28] was performed on a relatively small data set derived from the *C. elegans *genome. Also, the data sets considered in [32] are relatively small (around 300,000 examples, whereas more than 50,000,000 examples are nowadays readily available). In this study we therefore reevaluate the previous results on much larger data sets derived from the genomes of five model organisms, namely *Caenorhabditis elegans *("worm"), *Arabidopsis thaliana *("cress"), *Drosophila melanogaster *("fly"), *Danio rerio *("fish"), and *Homo sapiens *("human"). Building on our recent work on large scale kernel learning [37-40], we now are able to train and evaluate Support Vector Machines on such large data sets as is necessary for analyzing the whole human genome. In particular, we are able to show that increasing the number of training examples indeed helps to obtain a significantly improved performance, and thus will help to improve existing annotation (see, e.g. [3]). We train and evaluate SVMs on newly generated data sets using nested cross-validation and provide genome-wide splice site predictions for any occurring GT, GC and AG dimers, which will be furnished with posterior probability estimates for being true splice sites. We will show that the methods in some cases exhibit dramatic performance differences for the different data sets.

### Organization of the paper

The paper is organized as follows: In the next section we present the outcomes of (a) the comparison with the methods proposed in [12,29,32,36], (b) an assessment which window length should be used for classification and, finally, (c) a comparison of the large scale methods on the genome-wide data sets for the five considered genomes. After discussing our results, we also address the question about the interpretability of SVMs. Finally, in the Methods section, we describe the generation of our data sets, the details of cross-validation and model selection, different kernels, and visualizations method that we used in this study.

## Results and discussions

In this section we discuss experimental results we obtained with our methods for acceptor and donor splice site predictions for the five considered organisms.

Throughout the paper we measure our prediction accuracy in terms of area under the Receiver Operator Characteristic Curve (auROC) [41,42] and area under the Precision Recall Curve (auPRC) (e.g., [43]). (We omit to show the classification accuracy, as often more than 99% of the examples are negatively labeled. Thus, the simplest classifier, predicting -1 for all examples, already achieves 99% rendering the accuracy measure meaningless.) Note that for unbalanced data sets the area under the auROC can also be rather meaningless, since this measure is independent of class ratios and large auROC values may not necessarily indicate a good detection performance. The auPRC is a better measure for performance, if the class distribution is very unbalanced. However, it does depend on the class priors on the test set and hence is affected by sub-sampling the decoys, as happened with the data sets used in previous studies (NN269 in [32] contains about 4 times more decoy than true sites, DGSplicer in [32,36] about 140 times more; in contrast, in the genome scenario the ratio is one to 300 – 1000). In order to compare the results among the different data sets with different class sizes, we therefore also provide the auROC score which is not affected by sub-sampling.

### Pilot studies on small datasets

#### Performance on the NN269 and DGSplicer data sets

For the comparison of our SVM classifiers to the approaches proposed in [32,36], we first measure the performance of our methods on the four tasks used in [32] (see Methods for details). The approach in [32] is outperformed by a neural network approach proposed in [15]. However, we do not compare our methods to the latter method, since it already reaches its computational limits for the small datasets with only a few thousand short sequences (cf. [15], page 138) and hence is not suitable for large-scale genome-wide computations. On each task we trained SVMs with the *weighted degree kernel *(WD) [28], and the *weighted degree kernel with shifts *(WDS) [34]. On the NN269 Acceptor and Donor sets we additionally trained an SVM using the *locality improved kernel *(LIK) [21]; as it gives the weakest prediction performance and is computationally most expensive we exclude this model from the following investigations. As a benchmark method we also train higher order Markov Chains (MCs) (e.g. [44]) of "linear" structure and predict with the posterior log-odds ratio (cf. Methods section). Note that Position Specific Scoring Matrices (PSSM) are recovered as the special case of zeroth-order MCs. A summary of our results showing the auROC and auPRC scores is displayed in Table 1.

**Table 1:** Performance evaluation (auROC and auPRC scores) of six different methods on the NN269 and DGSplicer Acceptor and Donor test sets. MC denotes prediction with a Markov Chain, EBN the method proposed in [36], and MC-SVM the SVM based method described in [32] (similar to [21]).

	MC	EBN	MC-SVM	LIK	WD	WDS
**NN269**						
**Acceptor**						
*auROC*	96.78	-	96.74^†^	98.19	98.16	98.65
*auPRC*	88.41	-	88.33^†^	92.48	92.53	94.36
**Donor**						
*auROC*	98.18	-	97.64^†^	98.04	98.50	98.13
*auPRC*	92.42	-	89.57^†^	92.65	92.86	92.47

**DGSplicer**						
**Acceptor**						
*auROC*	97.23	95.91*	95.35*	-	97.50	97.28
*auPRC*	30.59	-	-	-	32.08	28.58
**Donor**						
*auROC*	98.34	96.88*	95.08*	-	97.84	97.47
*auPRC*	41.72	-	-	-	39.72	35.59

The remaining methods are based on SVMs using the locality improved kernel (LIK) [21], weighted degree kernel (WD) [28] and weighted degree kernel with shifts (WDS) [34]. The values marked with an asterisk were estimated from the figures provided in [32]. The values marked with † are from personal communication with the authors of [32].

We first note that the simple MCs perform already fairly well in comparison to the SVM methods. Surprisingly, we find that the MC-SVM proposed in [32] performs worse than the MCs. (We have reevaluated the results in [32] with the code provided by the authors and found that the stated false positive rate of their method is wrong by a factor of 10. We have contacted the authors for clarification and they published an erratum [45]. The results for MC-SVMs given in Table 1 are based on the corrected performance measurement.) As anticipated, for the two acceptor recognition tasks, EBN and MCs are outperformed by all kernel models which are performing all at a similar level. However, we were intrigued to observe that for the DGSplicer Donor recognition task, the MC based predictions outperform the kernel methods. For NN269 Donor recognition their performance is similar to the performance of the kernel methods.

**Figure 1:**
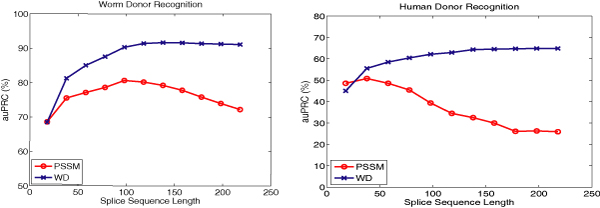
Comparison of classification performance of the weighted degree kernel based SVM classifier (WD) with the Markov chain based classifier (MC) on a subset of our *C. elegans Donor *and *Human Donor *data sets for sequences of varying length. For each length, we performed a full model selection on the training data in order to choose the best model. The performance on the test sets, measured through area under the Precision Recall Curve (auPRC), is displayed in percent.

There are at least two possible explanations for the strong performance of the MCs. First, the DGSplicer data set has been derived from the genome annotation, which in turn might have been obtained using a MC based gene finder. Hence, the test set may contain false predictions easier reproduced by a MC. Second, the window size for the DGSplicer Donor recognition task is very short and has been tuned in [36] to maximize the performance of their method (EBN) and might be suboptimal for SVMs. We investigated these hypotheses with two experiments:

• In the first experiment, we shortened the length of the sequences in DGSplicer Acceptor from 36 to 18 (with consensus AG at 8,9). We retrained the MC and WD models doing a full model selection on the shortened training data. We observe that on the shortened data set the prediction performance drops drastically for both MC and WD (by 60% relative) and that, indeed, the MC outperforms the WD method (to 12.9% and 9% auPRC, respectively).

• In a second experiment, we started with a subset of our new data sets generated from the genomes of worm and human which only uses EST or cDNA confirmed splice sites (see methods section). In the training data we used the same number of true and decoy donor sites as in the DGSplicer data set. For the test data we used the original class ratios (in order to allow a direct comparison to following experiments; cf. Table 2). Training and testing sequences were shortened from 218 nt in steps of 10 nt down to 18 nt (same as in the DGSplicer donor data set). We then trained and tested MCs and WD-SVMs for the sets of sequences of different length. Figure 1 shows the resulting values for the auPRC on the test data for different sequence lengths. For the short sequences, the prediction accuracies of MCs and SVMs are close for both organisms. For human donor sequences of length 18 MCs indeed outperform SVMs. With increasing sequence length, however, the auPRC of SVMs rapidly improves while it degrades for MCs. Recall that the short sequence length in the DGSplicer data was tuned through model selection for EBN, and thus the performance of the EBN method will degrade for longer sequences [36], so that we can safely infer that our methods would also outperform EBN for longer training sequences.

**Table 2:** Characteristics of the genome-wide data sets containing true and decoy acceptor and donor splice sites for our five model organisms.

	**Worm**		**Fly**		**Cress**		**Fish**		**Human**	
	Acceptor	Donor	Acceptor	Donor	Acceptor	Donor	Acceptor	Donor	Acceptor	Donor
Training total	1,105,886	1,744,733	1,289,427	2,484,854	1,340,260	2,033,863	3,541,087	6,017,854	6,635,123	9,262,241
Fraction positives	3.6%	2.3%	1.4%	0.7%	3.6%	2.3%	2.4%	1.5%	1.5%	1.1%
Evaluation total	371,897	588,088	425,287	820,172	448,924	680,998	3,892,454	10,820,985	10,820,985	15,201,348
Fraction positives	3.6%	2.3%	1.4%	0.7%	3.6%	2.3%	0.7%	0.4%	0.3%	0.2%
Testing total	364,967	578621	441,686	851,539	445,585	673,732	3,998,521	11,011,875	11,011,875	15,369,748
Fraction positives	3.6%	2.3%	1.4%	0.7%	3.5%	2.3%	0.7%	0.4%	0.3%	0.2%

The sequence length in all sets is 141 nt, for acceptor splice sequences the consensus dimer AG is at position 61, for donor GT/GC at position 81. The negative examples in training sets of fish and human were sub-sampled by a factor of three and five, respectively.

The results do not support our first hypothesis that the test data sets are enriched with MC predictions. However, the results confirm our second hypothesis that the poor performance of the kernel methods on the NN269 and DGSplicer donor tasks is due to the shortness of sequences. We also conclude that discriminative information between true and decoy donor sequences lies not only in the close vicinity of the splice site but also further away (see also the illustrations using *k*-mer scoring matrices below). Therefore, the careful choice of features is crucial for building accurate splice site detectors and if an appropriate window size is chosen, the WD kernel based SVM classifiers easily outperform previously proposed methods.

#### Comparison with SpliceMachine for cress and human

In this section we compare SpliceMachine [29] with the WD kernel based SVMs. SpliceMachine [46] is the current state-of-the art splice site detector. It is based on a linear SVM and outperforms the freely available GeneSplicer [47,12] by a large margin [29]. We therefore perform an extended comparison of our methods to SpliceMachine on subsets of the genome-wide datasets (cf. the results and methods sections). One fifth and one twenty-fifth of the data set was used each for training and for independent testing for cress and human, respectively. We downloaded the SpliceMachine feature extractor [48] to generate train and test data sets. Similar to the WD kernel, SpliceMachine makes use of positional information around the splice site. As it explicitly extracts these features it is however limited to a low order context (small *d*). In addition, SpliceMachine explicitly models coding-frame specific compositional context using tri- to hexamers. Note that this compositional information is also available to a gene finding system for which we are targeting our splicing detector. Therefore, in order to avoid redundancy, compositional information should ideally not be used to detect the *splicing signal*. Nevertheless, for comparative evaluation of the potential of our method, we augment our WD kernel based methods with 6 spectrum kernels [49] (order 3, 4, 5, each up- and downstream of splice site) and use the same large window sizes as were found out to be optimal in [29]. For cress acceptor [-85, +86], donor [-87, +124], and for human acceptor [-105, +146], donor [-107, +104]. For the WD kernel based SVMs, we fixed the model parameters *C *= 1 and *d *= 22, and for WDS we additionaly fixed the shift parameter *σ *= 0.5. For the SpliceMachine we performed an extensive model selection and found *C *= 10^-3 ^to be consistently optimal. We trained with *C *∈ {10^0^, 10^-1^, 10^-2^, 10^-3^, 5·10^-4^, 10^-4^, 10^-5^, 10^-6^, 10^-7^, 10^-8^}. Using these parameter settings we trained SVMs a) on the SpliceMachine features (SM), b) using the WD kernel (WD) c) using the WD kernel augmented by the 6 spectrum kernels (WDSP) d) using the WDS kernel (WDS) and e) using the WDS and spectrum kernels (WDSSP). Table 3 shows the area under the ROC and precision recall curve obtained in this comparison. Note that SpliceMachine always outperforms the WD kernel, but is in most cases inferior to the WDS kernel. Furthermore, complementing the WD kernels with spectrum kernels (methods WDSP and WDSSP) always improves precision beyond that of SpliceMachine. As this work is targeted at producing a splicing signal detector to be used in a gene finder, we will omit compositional information in the following genome-wide evaluations. To be fair, one can note that a WDS kernel using a very large shift is able to capture compositional information, and the same holds to some extend for the WD kernel when it has seen many training examples. It is therefore impossible to draw strong conclusions on whether window size and (ab)use of compositional features will prove beneficial when the splice site predictor is used as a module in a gene finder, which we hope is enabled by our work providing genome wide predictions.

**Table 3:** Performance evaluation (auROC and auPRC scores) of four different methods on a subset of the genome-wide cress and human datasets.

	SM	WD	WDSP	WDS	WDSSP
**Cress**					
**Acceptor**					
*auROC*	99.41	98.97	99.36	99.43	99.43
*auPRC*	91.76	84.24	90.64	92.01	92.09
**Donor**					
*auROC*	99.59	99.38	99.58	99.61	99.61
*auPRC*	93.34	88.62	93.42	93.68	93.87
**Human**					
**Acceptor**					
*auROC*	97.72	97.34	97.71	97.73	97.82
*auPRC*	50.39	42.77	50.48	51.78	54.12
**Donor**					
*auROC*	98.44	98.36	98.36	98.51	98.37
*auPRC*	53.29	46.53	54.06	53.08	54.69

The methods compared are SpliceMachine (SM), the weighted degree kernel (WD), the weighted degree kernel complemented with six spectrum kernels (WDSP), the weighted degree kernel with shifts (WDS), and the weighted degree kernel with shifts complemented by six spectrum kernels (WDSSP).

#### Performance for varying data size

**Figure 2:**
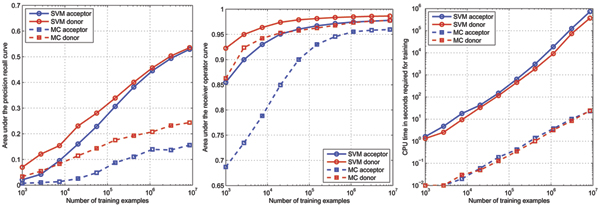
Comparison of the classification performance of the weighted degree kernel based SVM classifier (SVM) with the Markov chain based classifier (MC) for different training set sizes. The area under the Precision Recall Curve (auPRC; left) and the area under the Receiver Operator Curve (auROC; middle) are displayed in percent. On the right the CPU time in seconds needed to train the models is shown.

Figure 2 shows the prediction performance in terms of the auROC and auPRC of SVMs using the MC and the WD kernel on the human acceptor and donor splice data that we generated for this work (see the methods section) for varying training set sizes. For training we use up to 80% of all examples and the remaining examples for testing. MCs and SVMs were trained on sets of size varying between 1000 and 8.5 million examples. Here we sub-sampled the negative examples by a factor of five. We observe that the performance steadily increases when using more data for training. For SVMs, over a wide range, the auPRC increases by about 5% (absolute) when the amount of data is multiplied by a factor of 2.7. In the last step, when increasing from 3.3 million to 8.5 million examples, the gain is slightly smaller (3.2 – 3.5%), indicating the start of a plateau. Similarly MCs improve with growing training set sizes. As MCs are computationally a lot less demanding, we performed a full model selection over the model order and pseudo counts for each training set size. For the WD-SVM the parameters were fixed to the ones found optimal in the results section. Nevertheless MCs did constantly perform inferior to WD-SVMs. We may conclude that one should train using all available data to obtain the best results. If this is infeasible, then we suggest to only sub-sample the negatives examples in the training set, until training becomes computationally tractable. The class distribution in the test set, however, should never be changed unless explicitly taken into account in evaluation.

### Results on genome-wide data sets

Based on our preliminary studies, we now proceeded to design and train the genome-wide predictors. We first generated new *genome-wide *data sets for our five model organisms: worm, fly, cress, fish, and human. As our large-scale learning methods allow us to use millions of training examples, we included all available EST information from the commonly used databases. Since the reliability of the true and decoy splice sequences is crucial for a successful training and tuning, these data sets were produced with particular care; the details can be found in the Methods section. We arrived at training data sets of considerable size containing sequences of sufficient length (see Table 2). For fish and human the training datasets were sub-sampled to include only 1/3 and 1/5 of the negative examples, leading to a maximal training set size of 9 million sequences for human donor sites.

For a subsequent use in a gene finder system we aimed at producing unbiased predictions for *all *candidate splice sites, i.e. for all occurrences of the GT/GC and AG consensus dimer. For a proper model selection and in order to obtain unbiased predictions on the *whole *genome we employed nested five-fold cross-validation. We additionally estimated posterior probabilities in order to obtain interpretable and comparable scores for the outputs of the different SVM classifiers (see Methods for details). The results summarized in Table 4 are averaged values with standard deviation over the five different test partitions.

**Table 4:** Performance evaluation on the genome-wide data sets for worm, fly cress, fish, and human.

	**Worm**		**Fly**		**Cress**		**Fish**		**Human**	
	**Acc**	**Don**	**Acc**	**Don**	**Acc**	**Don**	**Acc**	**Don**	**Acc**	**Don**
**MC**										
auROC(%)	99.62 ± 0.03	99.55 ± 0.02	98.78 ± 0.10	99.12 ± 0.05	99.12 ± 0.03	99.44 ± 0.02	98.98 ± 0.03	99.19 ± 0.05	96.03 ± 0.09	97.78 ± 0.05
auPRC(%)	92.09 ± 0.28	89.98 ± 0.20	80.27 ± 0.76	78.47 ± 0.63	87.43 ± 0.28	88.23 ± 0.34	63.59 ± 0.72	62.91 ± 0.57	16.20 ± 0.22	24.98 ± 0.30

**WD**										
auROC(%)	99.77 ± 0.02	99.82 ± 0.01	99.02 ± 0.09	99.49 ± 0.05	99.37 ± 0.02	99.66 ± 0.02	99.36 ± 0.04	99.60 ± 0.04	97.76 ± 0.06	98.59 ± 0.05
auPRC(%)	95.20 ± 0.29	95.34 ± 0.10	84.80 ± 0.35	86.42 ± 0.60	91.06 ± 0.15	92.21 ± 0.17	85.33 ± 0.38	85.80 ± 0.46	52.07 ± 0.33	54.62 ± 0.54

**WDS**										
auROC(%)	99.80 ± 0.02	99.82 ± 0.01	99.12 ± 0.09	99.51 ± 0.05	99.43 ± 0.02	99.68 ± 0.02	99.38 ± 0.04	99.61 ± 0.04	97.86 ± 0.05	98.63 ± 0.05
auPRC(%)	95.89 ± 0.26	95.34 ± 0.10	86.67 ± 0.35	87.47 ± 0.54	92.16 ± 0.17	92.88 ± 0.15	86.58 ± 0.33	86.94 ± 0.44	54.42 ± 0.38	56.54 ± 0.57

Displayed are auROC and auPRC scores for acceptor and donor recognition tasks as archived by the MC method and two support vector machine approaches, one with the weighted degree kernel (WD) and one with the weighted degree kernewith shifts (WDS).

**Figure 3:**
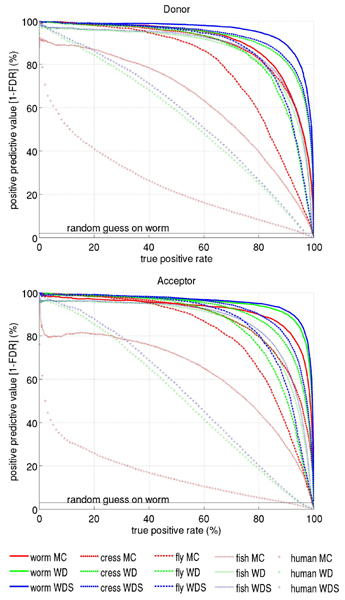
Precision Recall Curve for the three methods MC, WD, WDS estimated on the genome-wide data sets for worm, fly, cress, fish, and human in a nested cross-validation scheme. In contrast to the ROC the random guess in this plot corresponds to a horizontal line, that depends on the fraction of positive examples in the test set (e.g. 2% and 3% in the case of the worm acceptor and donor data sets, respectively).

Confirming our evaluations in the pilot studies, kernel methods outperform the MC methods in all eight classification tasks. Figure 3 displays the precision recall curves for all five organisms comparatively, Table 4 the corresponding auPRC scores. For worm, fly and cress the improvement in the performance accuracy for the SVM in comparison to MC lies in a similar range of 4–10% (absolute), both for donor and for acceptor tasks. However, for fish and especially for human the performance gain is considerable higher. For human, MCs only achieve 16% and 25% auPRC scores, whereas WDS reaches 54% and 57% for acceptor and donor recognition, respectively. The severe decrease in performance from worm to human for all classification methods in the auPRC score can partially be explained by the different fractions of positive examples observed in the test set. However, a weaker decline can also be observed in the auROC scores (also Table 4) which are independent of the class skew (e.g. for acceptor sites from 99.6% on worm to 96.0% on human for MC, and from 99.8% to 97.9% for WDS). The classification task on the human genome seems to be a considerably more difficult problem than the same one on the worm genome. We may speculate that this can be partially explained by a higher incidence of alternative splicing in the human genome. These sites usually exhibit weaker consensus sequences and are therefore more difficult to detect. Additionally, they often lead to mislabeled examples in the training and testing sets. Finally, it might also be due to the used protocol for aligning the sequences which may generate more false splice sites in human than in other organisms. This hypothesis is supported by the fact that the performance significantly increases, if one only considers cDNA confirmed genes (data not shown).

### Analysis of the learning result

One of the problems with kernel methods compared to probabilistic methods, such as Position Specific Scoring Matrices [50] or Interpolated Markov Models [11], is that the resulting decision function is hard to interpret and, hence, difficult to use in order to extract relevant biological knowledge from it (see also [51-53]). Here, we propose to use *k*-mer scoring matrices [3,54] to visualize the contribution of all (*k*-mer, sequence position) pairs to the final decision function of the SVM with WD-Kernel (cf. Methods section). We obtain a graphical representation from which it is possible to judge where in the sequence which substring lengths are of importance.

**Figure 4:**
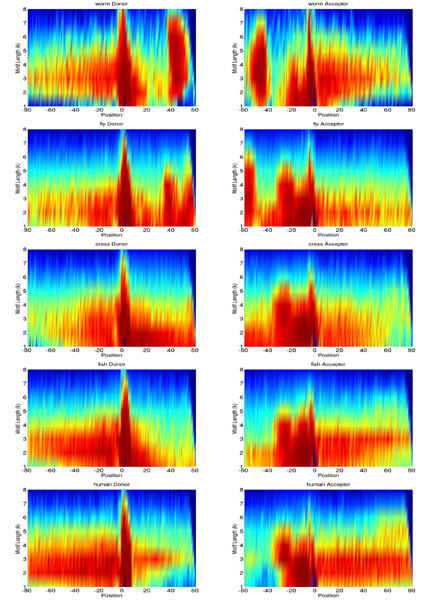
*k*-mer scoring matrices comparatively for worm, fly, cress, fish, and human. They depict the maximal position-wise contribution of all *k*-mers up to order 8 to the decision of the trained kernel classifiers, transformed into percentile values (cf. the section on interpreting the SVM classifier). Red values are highest contributions, blue lowest. Position 1 denotes the splice site and the start of the consensus dimer.

We plotted the *k*-mer scoring matrices corresponding to our trained models for the organisms comparatively in Figure 4, which shows the relative importance of substrings of a certain length for each position in the classified sequences. We can make a few interesting observations: For worm, fly, and potentially also cress there is a rather strong signal about 40–60 nt downstream of the donor and 40–60 nt upstream of the acceptor splice sites. These two signals are related to each other, since introns in these organisms are often only 50 nt long. Additionally, we find the region 20–30 nt upstream of the acceptor splice site of importance, which is very likely related to the branch point. In human it is typically located 20–50 nt upstream and exhibits the consensus CU(A/G)A(C/U), which matches the lengths of important *k*-mers in that region for human [55]. In worms, the branch point consensus seems shorter (3–4 nt) – confirming previous reports that the branch point is much weaker in worms. In fly and cress the branch point seems rather long (5–6 nt) and important for recognition of the splice site. Finally, note that the exon sequence carries a lot of discriminative information. The *k*-mers of most importance are of length three, relating to the coding potential of exons. Additionally, the periodicity observed for instance in cress is due to the reading frame. On the supplementary website we also provide a list of most discriminative *k*-mers for the two splice site recognition tasks.

## Conclusion

In this work we have evaluated several approaches for the recognition of splice sites in worm, fly, cress, fish, and human. In a first step we compared MCs, a Bayesian method (EBN) and SVM based methods using several kernels on existing data sets generated from the human genome. We considered the kernel used in [32] based on MCs, the locality improved kernel [21] and two variants of the weighted degree kernel [28,34]. We found that these existing data sets have limitations in that the sequences used for training and evaluation turn out to be too short for optimal discrimination performance. For SVMs we showed that they are able to exploit – albeit presumably weak – features as far as 80 nt away from the splice sites. In a comparison to SpliceMachine we could show that our approach perform favorably when complemented with compositional information. Using the protocol proposed in [3], we therefore generated new data sets for the five organisms. These data sets contain sufficiently long sequences and for human as many as 9 million training examples. Based on our previous work on large scale kernel learning [40], we were able to train SVM classifiers also on these rather big data sets. Moreover, we illustrated that the large amount of training data is indeed beneficial for significantly improving the SVM prediction performance, while MCs do not significantly improve when using much more training examples. We therefore encourage using as many examples for training as feasible to obtain the best generalization results.

For worm, fly and cress we were able to improve the performance by 4%–10% (absolute) compared to MCs. The biggest difference between the methods is observed for the most difficult task: acceptor and donor recognition on human DNA. The MCs reach only 16% and 25% auPRC, while SVMs achieve 54% and 57%, respectively. The drastic differences between organisms in the prediction performance scores can be understood as a consequence of the smaller fraction of positive examples and a higher incidence of alternative splicing in the human genome compared to the other genomes. For further comparative studies we provide and discuss *k*-mer scoring matrices elucidating which features are important for discrimination.

In order to facilitate the use of our classifiers for other studies, we provide whole genome predictions for the five organisms. Additionally, we offer an open-source stand-alone prediction tool allowing, for instance, the integration in other gene finder systems. The predictions, data sets and the stand-alone prediction tool are available for download on the supplementary website .

## Methods

### Data sets

#### NN269 and DGSplicer data sets

For the pilot study we use the NN269 and the DGSplicer data sets originating from [9] and [32], respectively. The data originates from [56] and the training and test splits can be downloaded from [46]. The data sets only include sequences with the canonical splice site dimers AG and GT. We use the same split for training and test sets as used in [32]. A description of the properties of the data set is given in Table 5.

**Table 5:** Characteristics of the NN269 and DGSplicer data sets containing true and decoy acceptor and donor splice sites derived from the human genome.

	**NN269**		**DGSplicer**	
	**Acceptor**	**Donor**	**Acceptor**	**Donor**
**Sequence length**	90	15	36	18
**Consensus positions**	AG at 69	GT at 8	AG at 26	GT at 10
**Train total**	5788	5256	322156	228268
**Fraction positives**	19.3%	21.2%	0.6%	0.8%
**Test total**	1087	990	80539	57067
**Fraction positives**	19.4%	21.0%	0.6%	0.8%

#### Worm, fly, cress, fish, and human

We collected all known ESTs from dbEST [57] (as of February 28, 2007; 346,064 sequences for worm, 514,613 sequences for fly, 1,276,130 sequences for cress, 1,168,572 sequences for fish, and 7,915,689 sequences for human). We additionally used EST and cDNA sequences available from wormbase [58] for worm, (file confirmed_genes.WS170) [59] for fly, (files na_EST.dros and na_dbEST.same.dmel) [60] for cress, (files cDNA_flanking_050524.txt and cDNA_full_reading_050524.txt) [61] for fish, (file Danio_rerio.ZFISH6.43.cdna.known.?? and [62] for fish and human (file dr_mgc_mrna.fasta for fish and hs_mgc_mrna.fasta for human). Using *blat *[63] we aligned ESTs and cDNA sequences against the genomic DNA (releases WS170, dm5, ath1, zv6, and hg18, respectively). If the sequence could not be unambiguously matched, we only considered the best hit. The alignment was used to confirm exons and introns. We refined the alignment by correcting typical sequencing errors, for instance by removing minor insertions and deletions. If an intron did not exhibit the consensus GT/AG or GC/AG at the 5' and 3' ends, we tried to achieve this by shifting the boundaries up to two base pairs (bp). If this still did not lead to the consensus, then we split the sequence into two parts and considered each subsequence separately. Then, we merged alignments if they did not disagree and if they shared at least one complete exon or intron.

In a next step, we clustered the alignments: In the beginning, each of the above EST and cDNA alignments were in a separate cluster. We iteratively joined clusters, if any two sequences from distinct clusters match to the same genomic location (this includes many forms of alternative splicing).

From the clustered alignments we obtained a compact splicing graph representation [64], which can be easily used to generate a list of positions of true acceptor and donor splice sites. Within the boundaries of the alignments (we cut out 10 nt at both ends of the alignments to exclude potentially undetected splice sites), we identified all positions exhibiting the AG, GT or GC dimer and which were not in the list of confirmed splice sites. The lists of true and decoy splice site positions were used to extract the disjoint training, validation and test sets consisting of sequences in a window around these positions. Additionally, we divided the whole genome into regions, which are disjoint contiguous sequences containing at least two complete genes; if an adjacent gene is less than 250 base pairs away, we merge the adjacent genes into the region. Genes in the same region are also assigned to the same cross-validation split. The splitting was implemented by defining a linkage graph over the regions and by using single linkage clustering. The splits were defined by randomly assigning clusters of regions to the split.

### Model selection and evaluation

To be able to apply SVMs, we have to find the optimal soft margin parameter *C *[18] and the kernel parameters. These are: For the LI-kernel, the degree *d *and window size *l*; for the WD kernel, the degree *d*; and for the WDS kernel, the degree *d *and the shift parameter *σ *(see the section on SVMs and kernels for details). For MCs we have to determine the order *d *of the Markov chain and the pseudocounts for the models of positive and the negative examples (see the posterior log-odds section). In order to tune these parameters we perform the cross-validation procedures described below.

#### NN269 and DGSplicer

The training and model selection of our methods for each of the four tasks was done separately by partial 10-fold cross-validation on the training data. For this, the training sets for each task are divided into 10 equally sized data splits, each containing the same number of splice sequences and the same proportion of true versus decoy sequences. For each parameter combination, we use only 3 out of the 10 folds, that is we train 3 times by using 9 out of the 10 training data splits and evaluate on the remaining training data split. Since the data is highly unbalanced, we choose the model with the highest average auPRC score on the three evaluation sets. This best model is then trained on the complete training data set. The final evaluation is done on the corresponding independent test sets (same as in [32]). The supplementary website includes tables with all parameter combinations used in model selection for each task and the chosen parameters.

#### Worm, fly, cress, fish, and human

The training and model selection of our methods for the five organisms on the acceptor and donor recognition tasks was done separately by 5-fold cross-validation. The optimal parameter was chosen by selecting the parameter combination that maximized the auPRC score. This model selection method was nested within 5-fold cross-validation for final evaluation of the performance. The reported auROC and auPRC are averaged scores over the five cross-validation splits. The supplementary website includes tables with all considered parameter combinations and the chosen parameters for each task. All splits were based on the basis of the clusters derived from EST and cDNA alignments, such that different splits come from random draws of the genome.

#### Performance measures

The sensitivity is defined as the fraction of correctly classified positive examples among the total number of positive examples, i.e. it equals the true positive rate *TPR *= *TP*/(*TP *+ *FN*). Analogously, the fraction *FPR *= *FP*/(*TN *+ *FP*) of negative examples wrongly classified as positive is called the false positive rate. Plotting *TPR *against *FPR *results in the Receiver Operator Characteristic Curve (ROC) [41,42]. Plotting the positive predictive value *PPV *= *TP*/(*FP *+ *TP*), i.e. the fraction of correct positive predictions among all positively predicted examples, against the *TPR*, one obtains the Precision Recall Curve (PRC) (see e.g., [43]). The area under the ROC and PRC are denoted by auROC and auPRC respectively.

#### Estimation of posterior probabilities

In order to provide an interpretable and comparable confidence score of the SVM predictions, we estimated the conditional likelihood *P*(*y *= 1|*f*(***x***)) of the true label *y *being positive for a given SVM output value *f*(***x***). To do this, we applied a piecewise linear function which was determined on the validation set (the same used for the classifier model selection). We used the *N *= 50 quantiles taken on the SVM output values as supporting points *φ*_*i*_, *i *= 1,...,*N*. For convenience, denote *φ*_0 _= -∞. For each point *φ*_*i *_the corresponding ${\hat{\pi }}_{i}$-value, which represents the empirical probability of being a true positive, was computed as ${\hat{\pi }}_{i}=\frac{n_{i}^{TP}}{n_{i}}$, where *n*_*i *_(*i *= 1,...,*N*) is the number of examples with output values *φ*_*i*-1 _≤ *f*(***x***) <*φ*_*i *_and $n_{i}^{TP}$ is the number of true splice sites in the same output range. Additionally, we determined the empirical cumulative probability as follows ${\hat{\pi }}_{i}^{c}=\left(\sum_{j=i}^{N}n_{j}^{TP}\right)/\left(\sum_{j=i}^{N}n_{j}\right)$. In order to obtain a smooth and strictly monotonically increasing probability estimate, we solve the following quadratic optimization problem:

$$\begin{array}{ll} \underset{\pi ,\pi^{c}\in {\mathbb{R}}_{+}^{N}}{\min} & \sum_{i=1}^{N}\left(s_{i}(\pi )+t_{i}(\pi^{c})\right)\\ \text{s}.\text{t}. & \pi_{i}\leq \pi_{i}^{c},\text{ for all }i=1,...,N\\ & \pi_{i}\leq \pi_{i+1}-\varepsilon ,\text{ for all }i=1,...,N-1\\ & \pi_{i}^{c}\leq \pi_{i+1}^{c}-\varepsilon \text{, for all }i=1,...,N-1, \end{array}$$

where *ε *= 10^-4 ^is a small constant ensuring that the functions are *strictly *monotonically increasing and $s_{i}(\pi )=\frac{n_{i}}{\sum_{j=1}^{N}n_{j}}{\left(\pi_{i}-{\hat{\pi }}_{i}\right)}^{2}$ and $t_{i}(\pi^{c})=\frac{\sum_{j=i}^{N}n_{j}}{\sum_{j=1}^{N}n_{j}}{\left(\pi_{i}^{c}-{\hat{\pi }}_{i}^{c}\right)}^{2}$ ensuring that big differences between the final and empirical estimates in ranges with many outputs are penalized stronger. Using the newly computed values *π*_1_,...,*π*_*N*_, we can compute for any output value *f*(***x***) the corresponding posterior probability estimate *P*(*y *= 1|*f*(***x***)) by linear interpolation

$$P(y=1|f(x))=\{\begin{array}{ll} \pi_{1} & \text{for }f(x)<\varphi_{1}\\ r(\varphi_{i},\varphi_{i+1}) & \text{for }\varphi_{i}\leq f(x)<\varphi_{i+1},\\ \pi_{N} & \text{for }f(x)\geq \varphi_{N} \end{array}$$

where $r(\varphi_{i},\varphi_{i+1})=\frac{\pi_{i+1}(f(x)-\varphi_{i})+\pi_{i}(\varphi_{i+1}-f(x))}{\varphi_{i+1}-\varphi_{i}}$. The cumulative posterior probability *P*^*c*^(*y *= 1|*f*(***x***)) is computed analogously. The above estimation procedure was performed separately for every classifier.

### Identifying splice sites

Machine learning binary classification methods aim at estimating a classification function *f *: X → {±1} using labeled training data from X × {±1} such that *g *will correctly classify unseen examples. In our case, the input space X will contain simple representations of sequences of length *N*, {*A*, *C*, *G*, *T*}^*N*^, while ±1 corresponds to true splice and decoy sites, respectively. We will use the posterior log-odds of a simple probabilistic model and SVMs using different kernels as classifiers as discussed below.

#### Posterior log-odds

The posterior log-odds of a probabilistic model with parameters ***θ ***are defined by

$$\begin{array}{lll} g(x) & := & \log(P(y=+1|x,\theta ))-\log(P(y=-1|x,\theta ))\\ & = & \log(P(x|\theta^{+}))-\log(P(x|\theta^{-}))+b, \end{array}$$

where *b *is a bias term. We use *f*(***x***) = sign(*g*(***x***)) for classification and Markov chains of order *d*

$$\begin{array}{lll} P(x|\theta^{\pm }) & = & P(x_{1},...,x_{N}|\theta^{\pm })\\ & = & P(x_{1},...,x_{d}|\theta^{\pm })\prod_{i=d+1}^{N}P(x_{i}|x_{i-1},...,x_{i-d},\theta^{\pm }) \end{array}$$

as for instance described in [44]. Each factor in this product has to be estimated in model training, i.e. one counts how often each symbol appears at each position in the training data conditioned on every possible *x*_*i*-1_,...,*x*_*i*-*d*_. Then for given model parameters ***θ ***we have

$$P(x|\theta^{\pm })=\theta_{0}^{\pm }(x_{1},...,x_{d})\prod_{i=d+1}^{N}\theta_{i}^{\pm }(x_{i},...,x_{i-d}),$$

where $\theta_{0}^{\pm }$ is an estimate for *P*(*x*_1_,...,*x*_*d*_) and *θ*_*i*_(*x*_*i*_,...,*x*_*i*-*d*_) an estimate for *P*(*x*_*i*_*|x*_*i*-1_,...,*x*_*i*-*d*_). As the alphabet has four letters, each model has (*N *- *d *+ 1)·4^*d*+1 ^parameters and the maximum likelihood estimate is given by:

$$\begin{array}{l} \theta_{0}(s_{1},...,s_{d})=\\ \frac{1}{m+\pi }\left(\sum_{k=1}^{m}I(s_{1}=x_{1}^{k}\wedge \cdots \wedge s_{d}=x_{d}^{k})+\pi \right)\\ \theta_{i}(s_{i},...,s_{i-d})=\\ \frac{\sum_{k=1}^{m}I(s_{i}=x_{i}^{k}\wedge \cdots \wedge s_{i-d}=x_{i-d}^{k})+\pi }{\sum_{k=1}^{m}I(s_{i}=x_{i-1}^{k}\wedge \cdots \wedge s_{i-d}=x_{i-d}^{k})+4\pi } \end{array}$$

where **I**(·) is the indicator function, *k *enumerates over the number of observed sequences *m*, and *π *is the commonly used pseudocount (a model parameter, cf. [44]) which is also tuned within the model selection procedure (cf. the model selection and evaluation section).

#### SVM and kernels for splice site detection

As the second method we use SVMs. The generated classification function can be written as

$$f(x)=\text{sign}\left(\sum_{i=1}^{m}y_{i}\alpha_{i}K(x_{i},x)+b\right),$$

where *y*_*i *_∈ {-1, +1} (*i *= 1,...,*m*) is the label of example ***x***_*i*_. The *α*_*i*_'s are Lagrange multipliers and *b *is the usual bias which are the results of SVM training [16]. The kernel K is the *key ingredient *for learning with SVMs.

In the following paragraphs we describe the kernels which are used in this study. They are all functions defined on sequences. In the following ***x ***= *x*_1_*x*_2_...*x*_*N *_denotes a sequence of length *N*.

**The locality improved (LI) kernel **has been proven useful in the context of translation initiation site (TIS) recognition [21]. Similar to the *polynomial kernel *of degree *d *for discrete input data, this kernel considers correlations of matches up to order *d*. In contrast to polynomial kernels however, the LI kernel only considers local subsequence correlations within a small window of length 2*l *+ 1 around a sequence position:

$${\text{win}}_{p}(x,x^{\prime })={\left(\frac{1}{2l+1}\sum_{j=-l}^{+l}I(x_{p+j}={x^{\prime }}_{p+j})\right)}^{d},$$

where *p *= *l *+ 1,...,*N *- *l*. These window scores are then summed up over the length of the sequence using a weighting *w*_*p *_which linearly decreases to both ends of the sequence, i.e. $w_{p}=\{\begin{array}{cc} p-l & p\leq N/2\\ N-p-l+1 & p>N/2 \end{array}$. Then we have the following kernel:

$$K(x,x^{\prime })=\sum_{p=l+1}^{N-l}w_{p}{\text{win}}_{p}(x,x^{\prime }).$$

The weighting allows one to emphasize regions of the sequence which are believed to be of higher importance; in our case this is the center, which is the location of the splice site. (Note that the definition of the LI kernel in [21] is slightly different from ours. Previously the weighting was inside the window and was not very effective. Moreover, the version presented here of the kernel can be computed 2*l *+ 1 times faster than the original one.)

**The weighted degree (WD) kernel **[28] uses a similar approach by counting matching subsequences ***u***_*δ*,*l*_(***x***) and ***u***_*δ*,*l*_(***x'***) between two sequences ***x ***and ***x'***, with ***u***_*δ*,*l*_(***x***) = *x*_*l*_*x*_*l*+1_...*x*_*l*+*δ*-1 _for all *l *and 1 ≤ *δ *≤ *d*. Here, *δ *denotes the order (length of the subsequence) to be compared. The WD kernel is defined as

$$K(x,x^{\prime })=\sum_{\delta =1}^{d}w_{\delta }\sum_{l=1}^{N-\delta +1}I(u_{\delta ,l}(x)=u_{\delta ,l}(x^{\prime })),$$

where we choose the weighting to be *w*_*δ *_= *d *- *δ *+ 1. This kernel emphasizes position dependent information and the weighting decreases the influence for higher order matches, which would anyway have a higher contribution due to all their matching subsequences. It can be computed very efficiently without even extracting and enumerating all subsequences of the sequences [40]. Note that this kernel is similar to the spectrum kernel as proposed by [49], with the main difference that the weighted degree kernel uses position specific information.

**The weighted degree kernel with shifts (WDS) **[34] is defined as

$$\begin{array}{c} K(x_{i},x_{j})=\sum_{\delta =1}^{d}w_{\delta }\sum_{l=1}^{N-\delta +1}\sum_{\begin{array}{c} s=0\\ s+l\leq N \end{array}}^{S(l)}\delta_{s}\mu_{\delta ,l,s,x_{i},x_{j}},\\ \mu_{\delta ,l,s,x_{i},x_{j}}=I(u_{\delta ,l+s}(x_{i})=u_{\delta ,l}(x_{j}))+I(u_{\delta ,l}(x_{i})=u_{\delta ,l+s}(x_{j})), \end{array}$$

where ***w***_*δ *_is as before, *δ*_*s *_= 1/(2(*s *+ 1)) is the weight assigned to shifts (in either direction) of extent *s*, and *S*(*l*) determines the shift range at position *l*. Here, we choose *S*(*l*) = *σ*|*l *- *l*_*c*_|, where *l*_*c *_is the position of the splice site. An efficient implementation for this kernel allowing large scale computations is described in [40].

For both the WD and WDS kernel we use the following normalization

$$\tilde{K}(x,x^{\prime })=\frac{K(x,x^{\prime })}{\sqrt{K(x,x)K(x^{\prime },x^{\prime })}}.$$

Training and evaluation of the SVMs and the MCs were performed using our shogun machine learning toolbox (cf. ) [38] in which efficient implementations of the aforementioned kernels can be found.

#### Interpreting the SVM classifier

Kernel methods are aimed directly at the classification task which is to *discriminate *between the true and decoy classes by learning a decision function separating the classes in an associated feature space. In contrast, *generative methods *like position weight matrices or Markov models are statistical models which represent the data under specific assumptions on the statistical structure and hence it is relatively straightforward to interpret their results. Although kernel methods outperform in many cases generative models, especially when the true statistical structure is more intricate than the assumed one, one of the main criticisms of kernel methods is the difficulty to directly interpret their decision function in a way that allows to gain biologically relevant insight. However, by taking advantage of our specific kernels and of their sparse representation, we are able to efficiently use the decision function of our SVMs in order to understand which *k*-mers at which positions are contributing the most in discriminating between true and decoy splice sites.

To see how this is possible, recall that, for SVMs, the resulting classifier can be written as a dot product between an ***α***-weighted linear combination of support vectors mapped into the feature space (which is often only implicitly defined via the kernel function) [18]:

$$f(x)=\underset{w}{\underset{︸}{\sum_{i=1}^{m}\alpha_{i}y_{i}\Phi (x_{i})}}\cdot \Phi (x)=\sum_{i=1}^{m}\alpha_{i}y_{i}K(x_{i},x).$$

In the case of sparse feature spaces, as with string kernels, one can represent ***w ***in a sparse form and then efficiently compute dot products between ***w ***and Φ(***x***) in order to speed up SVM training or testing [40]. This sparse representation comes with the additional benefit of providing us with means to interpret the SVM classifier. For *k*-mer based string kernels like the spectrum kernel, each dimension *w*_**u **_in ***w ***represents a weight assigned to that *k*-mer **u**. From the learned weighting one can thus easily identify the *k*-mers with highest absolute weight or above a given threshold *τ*: {***u ***| |*w*_*u*_| >*τ*}. Note that the total number of *k*-mers appearing in the support vectors is bounded by *dN*_*s*_*L *where *L *is the maximum length of the sequences $L=\max_{i=1,...,m}l_{x_{i}}$. This approach also works for the WD kernel (with and without shifts). Here a weight is assigned to each *k*-mer with 1 ≤ *k *≤ *d *at each position in the sequence. This allows us to generate the *k*-*mer importance matrices*, displayed in Figure 4, associated with our splice classifiers [54]. They display the weight which the SVM assigns to each *k*-mer at each position in the input sequence, i.e. given a SVM classifier trained with a WD kernel of degree *d *we extract the *k*-mers weightings for 1 ≤ *k *≤ $\tilde{d}$ starting at position *p *= 1,...,*N*, where we used *d *as selected in model selection and $\tilde{d}$ = 1,...,8. This leads to a weighting for $\tilde{d}$-mers **u **for each position in the sequence: *W*_**u**,*p*_, which may be summarized by $S_{\tilde{d},p}$ = max_**u**_(*W*_**u**,*p*_). We compute this quantity for $\tilde{d}$ = 1,...,8 leading to the two 8 × 141 matrices, which are transformed into percentile values and then displayed color-coded in Figure 4. Note that the above computation can be done efficiently using string index data structures implemented in *SHOGUN *and described in detail in [40].

## Competing interests

The authors declare that they have no competing interests.

## Authors' contributions

SS provided code for large scale kernel learning and helped carrying out the experiments. PP performed most experiments in the pilot study and drafted the manuscript. GS and JB performed the experiments on the genome-wide data sets and helped generating the data. GR conceived the experiments, generated the data sets and helped performing experiments. All authors contributed to the writing and critically revising the manuscript.
